# Association of Appropriate Empirical Antimicrobial Therapy With In-Hospital Mortality in Patients With Bloodstream Infections in the US

**DOI:** 10.1001/jamanetworkopen.2022.49353

**Published:** 2023-01-04

**Authors:** Tetsu Ohnuma, Shingo Chihara, Blair Costin, Miriam M. Treggiari, Raquel R. Bartz, Karthik Raghunathan, Vijay Krishnamoorthy

**Affiliations:** 1 Critical Care and Perioperative Population Health Research Unit, Department of Anesthesiology, Duke University Medical Center, Durham, North Carolina; 2 Section of Infectious Diseases, Department of Internal Medicine, Virginia Mason Medical Center, Seattle, Washington; 3 Department of Anesthesiology, Duke University, Durham, North Carolina; 4 Department of Anesthesia, Perioperative, and Pain Medicine, Brigham and Women’s Hospital, Boston, Massachusetts; 5 Anesthesia Service, Durham VA Medical Center, Durham, North Carolina

## Abstract

**Importance:**

Bloodstream infections (BSIs) are a major public health problem associated with high morbidity. Little evidence exists regarding the epidemiology of BSIs and the use of appropriate empirical antimicrobial therapy.

**Objective:**

To estimate the association between receipt of appropriate initial empirical antimicrobial therapy and in-hospital mortality.

**Design, Setting, and Participants:**

This retrospective cross-sectional study used data from the Premier Healthcare database from 2016 to 2020. The analysis included 32 100 adult patients (aged ≥18 years) with BSIs from 183 US hospitals who received at least 1 new systemic antimicrobial agent within 2 days after blood samples were collected during the hospitalization. Patients with polymicrobial infections were excluded from the analysis.

**Exposures:**

Appropriate empirical therapy was defined as initiation of at least 1 new empirical antimicrobial agent to which the pathogen isolated from blood culture was susceptible either on the day of or the day after the blood sample was collected.

**Main Outcomes and Measures:**

Multilevel logistic regression models were used to estimate the association between receipt of appropriate initial empirical antimicrobial therapy and in-hospital mortality for patients infected with gram-negative rods (GNRs), gram-positive cocci (GPC), and *Candida* species.

**Results:**

Among 32 100 patients who had BSIs and received new empirical antimicrobial agents, the mean (SD) age was 64 (16) years; 54.8% were male, 69.9% were non-Hispanic White, and in-hospital mortality was 14.3%. The most common pathogens were *Escherichia coli* (58.4%) and *Staphylococcus aureus* (31.8%). Among patients infected with *S aureus*, methicillin-resistant *S aureus* was isolated in 43.6%. The crude proportions of appropriate empirical therapy use were 94.4% for GNR, 97.0% for GPC, and 65.1% for *Candida* species. The proportions of appropriate therapy use for resistant organisms were 55.3% for carbapenem-resistant *Enterobacterales* species and 60.4% for vancomycin-resistant *Enterococcus* species. Compared with inappropriate empirical therapy, receipt of appropriate empirical antimicrobial therapy was associated with lower in-hospital risk of death for 3 pathogen groups (GNR: adjusted odds ratio [aOR], 0.52 [95% CI, 0.42-0.64]; GPC: aOR, 0.60 [95% CI, 0.47-0.78]; *Candida* species: aOR, 0.43 [95% CI, 0.21-0.87]).

**Conclusions and Relevance:**

In this cross-sectional study of patients hospitalized with BSIs, receipt of appropriate initial empirical antimicrobial therapy was associated with lower in-hospital mortality. It is important for clinicians to carefully choose empirical antimicrobial agents to improve outcomes in patients with BSIs.

## Key Points

**Question:**

Is the use of appropriate initial empirical antimicrobial therapy associated with lower in-hospital mortality in patients with bloodstream infections?

**Findings:**

In this cross-sectional study including 32 100 patients with bloodstream infections from 183 US hospitals, the crude proportions of appropriate empirical therapy use were 94.4% for gram-negative rods, 97.0% for gram-positive cocci, and 65.1% for *Candida* species, with lower proportions for resistant pathogens. Receipt of appropriate empirical antimicrobial therapy was associated with lower in-hospital mortality for patients infected with gram-negative rods, gram-positive cocci, and *Candida* species.

**Meaning:**

This study suggests that use of appropriate initial empirical antimicrobial therapy may have a role in inpatient hospital mortality.

## Teaser

This cross-sectional study assesses the association between receipt of appropriate initial empirical antimicrobial therapy and in-hospital mortality among adult patients with bloodstream infections in the US.

## Introduction

Bloodstream infections (BSIs) are recognized as a major public health problem worldwide.^1^ These infections are associated with short-term mortality ranging from 10% to 30%.^2,3,4^ Early adequate empirical antimicrobial treatment is the cornerstone of survival among patients with BSIs and sepsis.^5,6^ However, the delay in obtaining susceptibility of isolates as well as changes in antimicrobial resistance patterns make it difficult to choose appropriate antimicrobial agents.

Broad-spectrum antibiotics are recommended for patients with suspected severe infections to minimize the risk of undertreatment.^7,8,9,10^ However, the widespread use of broad-spectrum agents has been associated with antimicrobial resistance and frequent adverse events, such as allergic or hypersensitivity reactions, kidney injury, thrombocytopenia, *Clostridioides difficile* infection, and higher mortality.^7,11,12^ In contrast, in some settings, gram-negative organisms, such as carbapenem-resistant *Enterobacterales* and panresistant *Acinetobacter* species, have been spreading rapidly in the community, leading to a risk of undertreatment.^13^ Therefore, a delicate balance exists between overuse of broad-spectrum antibiotics and undertreatment of infections, both of which can lead to harm.

Previous studies^5,6,14^ looking at the association between appropriate empirical therapy and short-term outcomes reported conflicting results, primarily due to heterogeneity in the study populations and limitations in the study designs. Therefore, we designed a retrospective cross-sectional study of patients with BSIs to examine the characteristics of pathogens, the antibiotic resistance profile, and in-hospital mortality associated with receipt of appropriate empirical antimicrobial therapy. We hypothesized that the use of appropriate initial empirical therapy would be associated with a decreased risk of in-hospital death among patients with BSIs.

## Methods

### Study Design

We conducted a retrospective cross-sectional study using data from the Premier Healthcare database (Premier Inc) from 2016 to 2020. This study included patients from the subset of hospitals that reported laboratory and microbiological data. The Premier Healthcare database has information on patient demographic characteristics, hospital characteristics, date-stamped billing logs, and *International Statistical Classification of Diseases and Related Health Problems, Tenth Revision *(*ICD-10*) diagnosis and procedure codes. This study followed the Strengthening the Reporting of Observational Studies in Epidemiology (STROBE) guideline for cross-sectional studies. The study was approved by the Duke University Institutional Review Board and was determined to be exempt from requirements for informed consent because the data set was fully deidentified.

### Study Population

Patients were included if they were adults (aged ≥18 years) who had positive results from first blood cultures during the hospitalization and who received treatment with at least 1 new systemic antimicrobial agent within the initial 2 days of the blood sample collection. The index culture was defined as the first blood sample collection that was found to have positive results per case during the hospitalization. Patients with polymicrobial infections were excluded from the analysis.

### Characteristics of Pathogens and Site of Infection

The pathogen profile was constructed as a categorical variable with 3 levels according to the type of organism: gram-negative rods (GNRs), gram-positive cocci (GPC), and *Candida* species (eTable 1 in Supplement 1). We also assessed the incidence of resistant organisms, including methicillin-resistant *Staphylococcus aureus* (MRSA), vancomycin-resistant *Enterococcus* (VRE), extended-spectrum β-lactamase (ESBL) gram-negative organisms, carbapenem-resistant *Enterobacterales* (CRE; defined as resistant to imipenem, meropenem, doripenem, or ertapenem), and ceftriaxone-resistant gram-negative organisms (CTX-RO; including *Pseudomonas aeruginosa* and ESBL). Bloodstream infections due to coagulase-negative *Staphylococcus* species were considered as contaminants because it was not possible to determine whether they were definite infections. Bloodstream infections were considered hospital acquired if the first positive blood culture was obtained after 2 days of hospital admission.

We identified primary infection sites using *ICD-10* diagnosis codes (eTable 2 in Supplement 1).^12,15^ Patients could have more than 1 infection site because codes were not mutually exclusive.

### Antibiotic Susceptibility and Appropriate Initial Empirical Therapy

Appropriate empirical therapy was defined as initiation of at least 1 new empirical antimicrobial agent to which the pathogen isolated from blood culture was susceptible either on the day of or the day after the blood sample was collected. Nonsusceptibility to initial empirical therapy was defined as a microbial profile that was either resistant or intermediate. In cases in which susceptibilities to specific antimicrobial agents administered to patients were not listed in susceptibility data, we used interpretation tables developed by Rhee et al.^12^ Because susceptibility was generally not reported for other GNRs (eTable 1 in Supplement 1) and anaerobic pathogens, those organisms were not included in the analysis. We assessed antifungal susceptibilities for *Candida* species using Clinical and Laboratory Standards Institute guidelines.^16^ Patients were considered to have received appropriate empirical antifungal therapy if a *Candida* organism isolated from blood culture was covered by at least 1 antifungal agent administered.

### Outcome and Covariates

Our primary end point was in-hospital mortality during the index hospitalization. Patient demographic characteristics included age, sex, race and ethnicity (non-Hispanic Black, non-Hispanic White, or other [including American Indian or Alaska Native, Hispanic, Native Hawaiian or other Pacific Islander, non-Hispanic Asian, and any other racial or ethnic category other than non-Hispanic White or non-Hispanic Black]), payer category (managed care organization, Medicaid, Medicare, or other), comorbidities using the binary Elixhauser Comorbidity Index,^17^ transfer from another hospital, nosocomial infection, primary infection site, and hospital characteristics, including bed size (<200, 200-499, or ≥500), hospital teaching status, and hospital location (rural or urban). Categories for Hispanic, non-Hispanic Asian, non-Hispanic Black, non-Hispanic White, and non-Hispanic other or unknown race and ethnicity were provided by the Premier Healthcare database. We further combined the Hispanic, non-Hispanic Asian, and non-Hispanic other or unknown race and ethnicity categories into 1 group because we focused on the disparity between non-Hispanic Black and non-Hispanic White participants. Race and ethnicity were included as covariates because they were associated with antimicrobial drug use and outcomes. We also included data on intensive care unit admission, vasopressor use, mechanical ventilation, dialysis, creatinine, total bilirubin, and platelet count, which were collected within 2 days after blood sample collection.

### Statistical Analysis

Descriptive statistics were used to examine demographic and facility characteristics. Data were presented as means and SDs, medians and IQRs, or counts and percentages, as appropriate. Among patients with BSIs, we used descriptive statistics to estimate the frequency distribution of pathogens, antimicrobial resistance profiles, and antibiotic use. To estimate the association between receipt of appropriate initial empirical antimicrobial therapy and in-hospital mortality, we used multilevel multivariable logistic regression models with random intercepts for individual hospitals to account for clustering within hospitals. We fitted 3 models for GNRs, GPC, and *Candida* species separately because the measure of association differed among the 3 groups. The models were adjusted for covariates defined a priori that were selected on the basis of previous literature and expert opinion.^12,18^

In the sensitivity analysis, we examined appropriate empirical therapy use for each pathogen and each resistant organism. We conducted additional sensitivity analysis by removing markers of disease severity (intensive care unit admission, vasopressor use, mechanical ventilation, and dialysis) from covariates because they might have been mediators in the associations. We conducted a complete-case analysis because laboratory data within 2 days after blood sample collection had small percentages of missing information (0.3% for platelet count, 0.2% for creatinine, and 2.8% for total bilirubin).

For all analyses, 2-sided α <.05 was considered statistically significant. Analyses were performed using SAS software, version 9.4 (SAS Institute).

## Results

### Demographic and Clinical Characteristics

**Figure 1.**
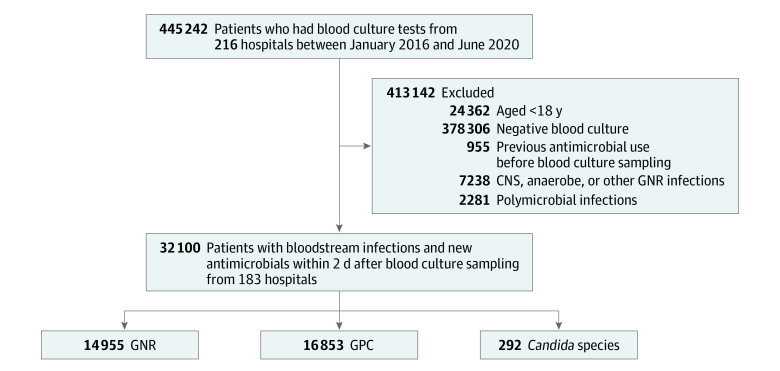
Study Cohort Flowchart CNS indicates coagulase-negative staphylococci; GNR, gram-negative rod; and GPC, gram-positive cocci.

Overall, 32 100 patients from 183 hospitals who had BSIs and received at least 1 new empirical antimicrobial agent met study eligibility criteria (Figure 1). The mean (SD) age of the patients in the total sample was 64 (16) years; 54.8% were male, 69.9% were non-Hispanic White, and the in-hospital mortality rate was 14.3%. Overall, 46.6% of patients had positive results for GNRs, 52.5% had positive results for GPC, and 0.9% had positive results for *Candida* species. The demographic and clinical characteristics of patients with BSIs are shown in Table 1. The common sites of infection were pulmonary and genitourinary across the groups. Congestive heart failure, diabetes, and kidney disease were the most common comorbidities in this study cohort. Vancomycin was the most frequently used initial empirical therapy across the groups, followed by cephalosporin and penicillin. Overall, 94.2% of BSIs were not hospital-acquired infections.

**Table 1.** Characteristics of Patients With Bloodstream Infections

Characteristic	Patients, No. (%)					
	GNR		GPC		*Candida* species	
	Inappropriate therapy (n = 841)	Appropriate therapy (n = 14 114)	Inappropriate therapy (n = 512)	Appropriate therapy (n = 16 341)	Inappropriate therapy (n = 102)	Appropriate therapy (n = 190)
Age, median (IQR), y	69 (60-79)	69 (58-78)	67 (55-77)	63 (51-73)	65 (54-77)	62 (48-72)
Sex						
Male	419 (49.8)	6792 (48.1)	302 (59.0)	9920 (60.7)	57 (55.9)	100 (52.6)
Female	422 (50.2)	7328 (51.9)	210 (41.0)	6421 (39.3)	45 (44.1)	90 (47.4)
Race and ethnicity						
Non-Hispanic Black	99 (11.8)	1594 (11.3)	82 (16.0)	2014 (12.3)	18 (17.6)	28 (14.7)
Non-Hispanic White	556 (66.1)	9507 (67.4)	361 (70.5)	11 787 (72.1)	73 (71.6)	143 (75.3)
Other^a^	186 (22.1)	3013 (21.3)	69 (13.5)	2540 (15.5)	11 (10.8)	19 (10.0)
Payer category						
Managed care organization	88 (10.5)	1855 (13.1)	53 (10.4)	2246 (13.7)	10 (9.8)	31 (16.3)
Medicaid	96 (11.4)	1566 (11.1)	89 (17.4)	2970 (18.2)	19 (18.6)	38 (20.0)
Medicare	599 (71.2)	9435 (66.8)	328 (64.1)	9321 (57.0)	67 (65.7)	108 (56.8)
Other	58 (6.9)	1258 (8.9)	42 (8.2)	1804 (11.0)	6 (5.9)	13 (6.8)
Comorbidities						
CHF	244 (29.0)	3349 (23.7)	170 (33.2)	5031 (30.8)	31 (30.4)	54 (28.4)
COPD	215 (25.6)	2847 (20.2)	135 (26.4)	3841 (23.5)	28 (27.5)	50 (26.3)
Diabetes	343 (40.8)	5654 (40.1)	235 (45.9)	7446 (45.6)	38 (37.3)	81 (42.6)
Kidney disease	256 (30.4)	3882 (27.5)	161 (31.4)	4953 (30.3)	28 (27.5)	55 (28.9)
PVD	69 (8.2)	1060 (7.5)	45 (8.8)	1878 (11.5)	11 (10.8)	16 (8.4)
Neurological disorder	151 (18.0)	2237 (15.8)	74 (14.5)	2285 (14.0)	21 (20.6)	33 (17.4)
Liver disease	88 (10.5)	1712 (12.1)	60 (11.7)	2075 (12.7)	15 (14.7)	23 (12.1)
Cancer	110 (13.1)	1813 (12.8)	58 (11.3)	1241 (7.6)	16 (15.7)	33 (17.4)
Transfer from another hospital	79 (9.4)	67 (0.5)	50 (9.8)	1429 (8.7)	26 (25.5)	6 (3.2)
Nosocomial infection	69 (8.2)	804 (5.7)	30 (5.9)	943 (5.8)	6 (5.9)	20 (10.5)
Site of infection						
Pulmonary	203 (24.1)	2886 (20.4)	144 (28.1)	5267 (32.2)	26 (25.5)	62 (32.6)
Genitourinary	340 (40.4)	6155 (43.6)	89 (17.4)	3175 (19.4)	19 (18.6)	46 (24.2)
Intra-abdominal	76 (9.0)	1600 (11.3)	31 (6.1)	635 (3.9)	11 (10.8)	28 (14.7)
Skin or soft tissue	87 (10.3)	974 (6.9)	93 (18.2)	4449 (27.2)	5 (4.9)	13 (6.8)
Bone or joint	25 (3.0)	304 (2.2)	42 (8.2)	2208 (13.5)	3 (2.9)	7 (3.7)
Other	262 (31.2)	3478 (24.6)	188 (36.7)	8696 (53.2)	70 (68.6)	171 (90.0)
Organ dysfunction						
Vasopressor	384 (45.7)	6093 (43.2)	153 (29.9)	5340 (32.7)	53 (52.0)	85 (44.7)
Mechanical ventilation	210 (25.0)	3032 (21.5)	118 (23.0)	3729 (22.8)	41 (40.2)	72 (37.9)
Dialysis	90 (10.7)	1265 (9.0)	55 (10.7)	2055 (12.6)	9 (8.8)	27 (14.2)
Maximum creatinine, median (IQR), mg/dL	1.7 (1.1-2.9)	1.7 (1.1-2.8)	1.6 (1.1-3.0)	1.5 (1.0-2.9)	2.0 (1.1-3.1)	1.9 (1.1-3.5)
Maximum total bilirubin, median (IQR), mg/dL	0.9 (0.6-1.6)	1.0 (0.6-1.9)	0.7 (0.5-1.3)	0.9 (0.5-1.4)	0.7 (0.4-1.4)	0.8 (0.5-1.8)
Minimum platelet count, median (IQR), 10^9^ cells/L	152 (98-211)	132 (84-188)	178 (118-263)	162 (101-236)	170 (90-246)	141 (80-209)
ICU admission	571 (67.9)	9898 (70.1)	286 (55.9)	9150 (56.0)	67 (65.7)	121 (63.7)
Antimicrobial profile						
Duration of antimicrobial therapy, median (IQR), d^b^	7 (4-11)	7 (5-11)	8 (4-13)	9 (6-15)	7 (3-11)	7 (4-12)
Medication						
Cephalosporin	455 (54.1)	9008 (63.8)	287 (56.1)	10 334 (63.2)	52 (51.0)	116 (61.1)
Penicillin	347 (41.3)	7478 (53.0)	184 (35.9)	8657 (53.0)	59 (57.8)	90 (47.4)
Carbapenem	144 (17.1)	4291 (30.4)	42 (8.2)	1787 (10.9)	15 (14.7)	47 (24.7)
Quinolone	104 (12.4)	2330 (16.5)	75 (14.6)	1825 (11.2)	11 (10.8)	22 (11.6)
Vancomycin	505 (60.0)	8053 (57.1)	178 (34.8)	14 772 (90.4)	68 (66.7)	148 (77.9)
Antifungal agent	46 (5.5)	687 (4.9)	21 (4.1)	566 (3.5)	8 (7.8)	190 (100)
Hospital characteristics						
Teaching hospital	437 (52.0)	6386 (45.2)	252 (49.2)	8448 (51.7)	53 (52.0)	126 (66.3)
Rural hospital	135 (16.1)	2163 (15.3)	63 (12.3)	2117 (13.0)	10 (9.8)	21 (11.1)
Bed size						
<200	186 (22.1)	3475 (24.6)	107 (20.9)	3468 (21.2)	18 (17.6)	29 (15.3)
200-499	371 (44.1)	5916 (41.9)	249 (48.6)	6847 (41.9)	36 (35.3)	73 (38.4)
≥500	284 (33.8)	4723 (33.5)	156 (30.5)	6026 (36.9)	48 (47.1)	88 (46.3)
Hospital LOS, median (IQR), d	7 (4-12)	7 (4-11)	9 (4-14)	9 (6-16)	8 (2-15)	10 (5-18)
In-hospital mortality	174 (20.7)	1808 (12.8)	108 (21.1)	2412 (14.8)	35 (34.3)	45 (23.7)

Abbreviations: CHF, congestive heart failure; COPD, chronic obstructive pulmonary disease; GNR, gram-negative rod; GPC, gram-positive cocci; ICU, intensive care unit; LOS, length of stay; PVD, peripheral vascular disease.

SI conversion factors: To convert creatinine from milligrams per deciliter to micromoles per liter, multiply by 88.4; to convert total bilirubin from milligrams per deciliter to micromoles per liter, multiply by 17.104.

^a^ Other races and ethnicities include American Indian or Alaska Native, Asian, Hispanic, Native Hawaiian or other Pacific Islander, and any other racial or ethnic category other than non-Hispanic White or non-Hispanic Black. If Hispanic ethnicity information was unknown, patients were considered non-Hispanic, and information on race alone was used to classify race and ethnicity.

^b^ The duration of antimicrobial therapy was calculated using all antimicrobial agents that were administered to patients.

### Pathogen Characteristics

**Figure 2.**
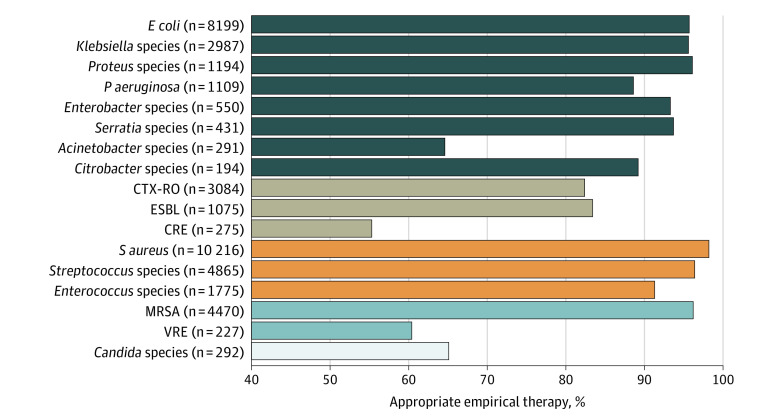
Number of Patients Infected With Pathogens and Proportions of Appropriate Empirical Antimicrobial Therapy by Pathogen CRE indicates carbapenem-resistant *Enterobacterales*; CTX-RO, ceftriaxone-resistant gram-negative organism; *E coli*, *Escherichia coli*; ESBL, extended-spectrum β-lactamase–producing gram-negative organism; MRSA, methicillin-resistant *Staphylococcus aureus*; *P aeruginosa*, *Pseudomonas aeruginosa*; *S aureus*, *Staphylococcus aureus*; and VRE, vancomycin-resistant *Enterococcus*.

The distribution of pathogens in patients with BSIs is summarized in Figure 2. Among 14 030 GNRs, *Escherichia coli* was the most common pathogen (58.4%), followed by *S aureus* (31.8%), *Klebsiella* species (21.3%), *Proteus* species (8.5%), and *P aeruginosa* (7.9%). Other organisms were less frequently represented. Of the 32 100 patients included in the study, CTX-RO was isolated in 9.6%, ESBL in 3.3%, and CRE in 0.9%. The frequency of MRSA in all BSIs was 13.9%, and the frequency of MRSA among patients infected with *S aureus* was 43.6%. The prevalence of VRE in all BSIs was 0.7%.

Overall, the proportions of appropriate empirical therapy use were 94.4% for GNRs, 97.0% for GPC, and 65.1% for *Candida* species. The proportions of appropriate empirical antimicrobial therapy varied by pathogen, ranging from 55.3% for patients infected with CRE to 98.2% for patients infected with *S aureus*. The proportions of appropriate empirical therapy for resistant organisms were 55.3% for carbapenem-resistant *Enterobacterales* species and 60.4% for vancomycin-resistant *Enterococcus* species. *Acinetobacter* species had the lowest proportion of appropriate empirical antimicrobial therapy (64.6%) among nonresistant GNRs. The proportions of appropriate empirical antimicrobial therapy for nonresistant pathogens were generally higher than those for resistant pathogens (Figure 2). For *Candida* species, among 102 patients who received inappropriate empirical antimicrobial therapy, the proportion who did not receive empirical antifungal therapy was 92.2%.

### Association of Empirical Appropriate Therapy With In-Hospital Mortality

**Figure 3.**
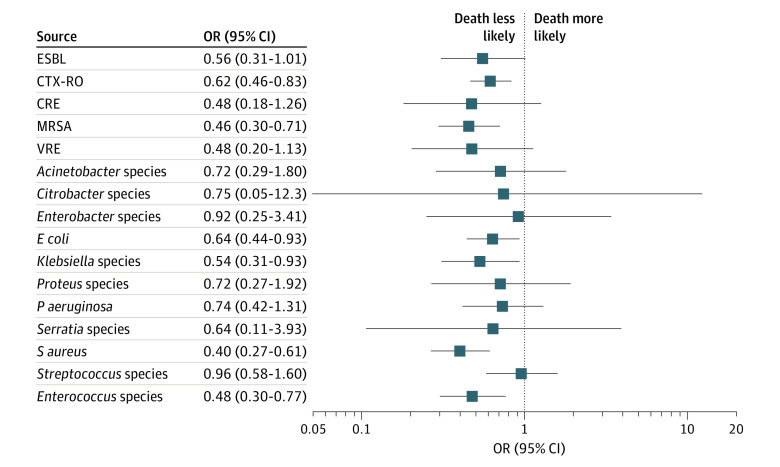
Adjusted Odds of In-Hospital Death Associated With Appropriate Empirical Antimicrobial Therapy by Pathogen CRE indicates carbapenem-resistant *Enterobacterales*; CTX-RO, ceftriaxone-resistant gram-negative organism; *E coli*, *Escherichia coli*; ESBL, extended-spectrum β-lactamase–producing gram-negative organism; MRSA, methicillin-resistant *Staphylococcus aureus*; OR, odds ratio; *P aeruginosa*, *Pseudomonas aeruginosa*; *S aureus*, *Staphylococcus aureus*; and VRE, vancomycin-resistant *Enterococcus*.

The crude in-hospital mortality rates for the appropriate empirical therapy group compared with the inappropriate empirical therapy group were 1808 of 14 114 (12.8%) vs 174 of 841 (20.7%) for GNR, 2412 of 16 341 (14.8%) vs 108 of 512 (21.1%) for GPC, and 45 of 190 (23.7%) vs 35 of 102 (34.3%) for *Candida* species (Table 2). In multivariable multilevel logistic regression models, compared with inappropriate empirical therapy, receipt of appropriate empirical antimicrobial therapy was associated with lower in-hospital risk of death for the 3 pathogen groups (GNR: adjusted odds ratio [aOR], 0.52 [95% CI, 0.42-0.64]; GPC: aOR, 0.60 [95% CI, 0.47-0.78]; and *Candida* species: aOR, 0.48 [95% CI, 0.23-0.99]) (Figure 3). The sensitivity analysis removing markers of disease severity from covariates yielded similar results (GNR: aOR, 0.52 [95% CI, 0.43-0.64]; GPC: aOR, 0.65 [95% CI, 0.51-0.82]; *Candida* species: aOR, 0.43 [95% CI, 0.21-0.87]).

**Table 2.** In-Hospital Mortality Associated With Receipt of Appropriate vs Inappropriate Initial Empirical Antimicrobial Therapy^a^

Type of BSI	Patients, No./total No. (%)		OR (95% CI)	
	Appropriate therapy	Inappropriate therapy	Unadjusted	Adjusted
GNR	1808/14 114 (12.8)	174/841 (20.7)	0.57 (0.48-0.68)	0.52 (0.42-0.64)
GPC	2412/16 341 (14.8)	108/512 (21.1)	0.65 (0.52-0.82)	0.60 (0.47-0.78)
*Candida* species	45/190 (23.7)	35/102 (34.3)	0.57 (0.32-1.01)	0.48 (0.23-0.99)

Abbreviations: BSI, bloodstream infection; GNR, gram-negative rod; GPC, gram-positive cocci; OR, odds ratio.

^a^ To assess the association of receipt of appropriate empirical therapy with in-hospital mortality, models were fitted among patients with GNR, GPC, and *Candida* species. Each model was adjusted for age, sex, race and ethnicity, payer category, comorbidities, transfer from another hospital, nosocomial infection, primary infection site, hospital characteristics, intensive care unit admission, vasopressor use, mechanical ventilation, dialysis, and laboratory data.

In the sensitivity analysis for specific types of pathogens, in-hospital risk of death associated with appropriate empirical therapy was lower for patients infected with *E coli* (aOR, 0.64; 95% CI, 0.44-0.93), *Klebsiella* species (aOR, 0.54; 95% CI, 0.31-0.93), CTX-RO (aOR, 0.62; 95% CI, 0.46-0.83), *S aureus* (aOR, 0.40; 95% CI, 0.27-0.61), MRSA (aOR, 0.46; 95% CI, 0.30-0.71), and *Enterococcus* species (aOR, 0.48; 95% CI, 0.30-0.77).

## Discussion

In this retrospective cross-sectional study investigating patients with BSIs across 183 US hospitals from 2016 to 2020, we found that the most frequent gram-negative organisms were *E coli* and *Klebsiella* species, and the most frequent gram-positive organisms were *S aureus* and *Streptococcus* species. The majority of patients with BSIs did not have hospital-acquired infections. The proportions of initial appropriate empirical therapy varied among pathogens but were generally high for nonresistant pathogens. Receipt of initial appropriate empirical therapy was associated with lower in-hospital mortality for patients infected with GNRs, GPC, and *Candida* species. These data have implications for the use of empirical antimicrobial agents among patients with BSIs.

We found that the proportions of appropriate empirical therapy were generally high (94.4% for GNRs and 97.0% for GPC) and better than the proportions reported in previous studies.^5,12,18,19,20,21^ A meta-analysis of 27 studies^5^ found that the proportion of inappropriate empirical antibiotic use for severe infection was more than 50% in approximately one-half of the studies. On the other hand, appropriate initial empirical therapy use varied among pathogens and was generally lower for resistant organisms.^5^ Our work extends the findings of a multicenter electronic health record–based study^18^ including 21 608 patients with BSIs using the Cerner database from 2005 to 2014, which reported that patients infected with resistant organisms were more likely to receive discordant empirical antibiotic therapy. Although the implementation of antibiotic stewardship programs, the advancement of diagnostic tools, and the development of guidelines might have had implications for the improvement in appropriate empirical therapy use observed in our study, selecting appropriate empirical therapy remains a challenge, particularly for resistant pathogens. Our findings are consistent with 2 population health studies^12,18^ investigating BSIs and culture-positive sepsis using the Cerner database, which suggested that undertreatment was associated with higher mortality. In contrast, several studies^19,20,21^ have revealed that inadequate empirical antibiotic therapy was not associated with mortality. This discrepancy might be explained by the small size of the studied population or limitations in the study design, such as the definition of inappropriate empirical antibiotic therapy.

Advances in diagnostic approaches, including molecular diagnostic testing as well as implementation of antimicrobial stewardship programs, may play an important role in ensuring that patients receive adequate treatment in a timelier fashion than in the past.^22,23,24^ Identifying potential risk factors for resistant pathogens might help improve the choice of correct empirical antibiotic therapy in patients with a high likelihood of infection with resistant pathogens.^7^ A judicious selection of broad empirical antimicrobial agents to treat BSIs is essential, but a comprehensive approach would also be warranted to further improve outcomes.

Our study found a low incidence of candidemia but high use of discordant empirical antifungal therapy. It is important to note that the lack of timely diagnoses for candidemia makes it difficult to determine whether empirical antifungal therapy needs to be started. Consistent with previous studies reporting that candidemia in patients with severe disease was associated with an increased risk of death,^16,25^ we found that use of inadequate empirical antifungal therapy was associated with higher in-hospital mortality. Guidelines have recommended considering empirical antifungal therapy for the treatment of patients with risk factors for fungal infections, although clinical studies on the effectiveness of such empirical strategies are limited.^7,16^ A randomized clinical trial^26^ including 260 patients who were critically ill with sepsis did not find that receipt of empirical antifungal therapy improved 28-day fungal infection–free survival. Thus, the decision to initiate empirical antifungal therapy needs to be made individually based on an assessment of risk factors for fungal infection.

### Limitations

This study has limitations inherent to a retrospective design. First, the study cohort was not necessarily representative of all US hospitals; therefore, our findings might not be generalizable to other hospitals. Second, we were unable to obtain specific illness severity scores, allowing for the possibility of residual confounding in our data. However, we were able to include markers of disease severity, such as vasopressor use, mechanical ventilation, and laboratory data representative of organ dysfunction as well as many other confounders in our regression models, which might mitigate the consequences of unmeasured confounding. Third, detailed information on the time to adequate source control or the time to antimicrobial administration was unavailable. Fourth, we were unable to identify CRE using the Centers for Disease Control and Prevention (CDC) definition because information on carbapenemases was lacking. This issue could have led to underestimation of those organisms. Similarly, we were unable to use the CDC National Healthcare Safety Network definition to determine whether patients had a BSI due to coagulase-negative *Staphylococcus* species because vital signs and notes were unavailable. Moreover, we considered third-generation cephalosporins to be susceptible if they were phenotypically susceptible to organisms with chromosomal AmpC, such as *Enterobacter*, *Klebsiella aerogenes*, *Citrobacter*, and *Serratia*. However, we might have missed some AmpC-producing organisms that were initially susceptible and might become resistant within 3 to 4 days after initiation of therapy, which might have had implications for the outcome. Fifth, our data sets did not allow us to account for appropriate doses of prescribed antimicrobial agents. Sixth, although we included a large sample of patients hospitalized for BSIs, the sample size might still have been too small to detect mortality differences for specific pathogens. It is also possible that some blood cultures with positive results for pathogens could have been contaminated (eg, 1 set of α-hemolytic *Streptococcus*). However, patients had septic syndrome associated with their cultures and were therefore likely to have true BSIs.

## Conclusions

In this multicenter cross-sectional study of patients with BSIs, the incidence of appropriate initial empirical therapy was 94.4% for GNRs, 97.0% for GPC, and 65.1% for *Candida* species. Receipt of appropriate initial empirical therapy for GNRs, GPC, and *Candida* species was associated with lower in-hospital mortality. Patients infected with resistant pathogens were more likely to receive inappropriate empirical therapy. Given these findings, it is important for clinicians to carefully choose empirical antimicrobial agents to improve outcomes in patients with BSIs.

## Notes

Supplement 1. **eTable 1.** Categorization of Pathogens **eTable 2.** Classification of Infections With Corresponding *ICD-10* Codes

Supplement 2.
Data Sharing Statement
