# Supplementary material for: Association of Appropriate Empirical Antimicrobial Therapy With In-Hospital Mortality in Patients With Bloodstream Infections in the US
Source: JAMA Netw Open. 2023 Jan 4;6(1):e2249353. doi: 10.1001/jamanetworkopen.2022.49353 (PMC9857618; doi:10.1001/jamanetworkopen.2022.49353)
Supplement: Supplement 1. — eTable 1. Categorization of Pathogens eTable 2. Classification of Infections With Corresponding ICD-10 Codes [file jamanetwopen-e2249353-s001.pdf]

Supplemental Online Content

Ohnuma T, Chihara S, Costin B, et al. Association of appropriate empirical antimicrobial therapy with in-hospital mortality in patients with bloodstream infections in the US. *JAMA Netw Open*. 2023;6(1):e2249353. doi:10.1001/jamanetworkopen.2022.49353

eTable 1. Categorization of Pathogens

eTable 2. Classification of Infections With Corresponding *ICD-10* Codes

This supplemental material has been provided by the authors to give readers additional information about their work.

© 2023 Ohnuma T et al. *JAMA Network Open*.

eTable 1. Categorization of Pathogens

Gram-negative organism	*Acinetobacter* species, *Citrobacter* species, *Enterobacter* species, *Escherichia coli*, *Klebsiella* species, *Proteus* species, *Pseudomonas aeruginosa*, and *Serratia* species, and other gram-negative organisms.
Gram-positive organism	*Staphylococcus aureus*, *Streptococcus* species, and *Enterococcus* species, and other gram-positive organisms.
Candida	*Candida albicans, Candida glabrata*, *Candida parapsilosis*, *Candida tropicalis, Candida krusei*, and other *Candida* species.

Coagulase-negative *Staphylococcus* species were considered as contaminants. Anaerobes were not included in the analysis.

© 2023 Ohnuma T et al. *JAMA Network Open*.

eTable 2. Classification of Infections With Corresponding *ICD-10* Codes

Genitourinary	N10, N30.0, N30.8, N30.9, N39.0, N41.0, N41.2, N41.3, N41.8, N45, N70-N77
Intra-Abdominal	K10.2, K11.3, K12.2, K35, K57.0, K57.2, K57.4, K57.8, K61, K63.0, K63.1, K65, K75.0, K75.1, K81.0, K83.0,
Pulmonary	J01, J02, J03, J04, J06, J09-J18, J20-J22, J36, J39.0, J39.1, J44.0, J47.0, J85-J86, J98.50
Bone/Joint	M00, M01, M46.2, M46.3, M46.5, M60.0, M72.6, M86,
Skin/Soft Tissue	L00, L01, L02, L03, L04, L05, L08, L88,
Other	A00-A09, A15-A19, A20-A28, A30-39, A42-A49, A50-A64, A70-A74, A92-A99, B00-B02, B05, B06, B15-B19, B25-B34, B37-B40, B44, B49, B50-B64, B99,<br>G00-G02, G04-G08,<br>H05.0, H44.0,<br>H60.2, H70.0,<br>I30.1, I33.0, I40.0,<br>O03.0, O03.5, O04.0, O05.0, O06.0, O07.0, O08.0, O23, O41.1, O75.3, O85, O86,<br>T79.3, T82-T85

© 2023 Ohnuma T et al. *JAMA Network Open*.
