# Supplementary material for: Association of Appropriate Empirical Antimicrobial Therapy With In-Hospital Mortality in Patients With Bloodstream Infections in the US
Source: JAMA Netw Open. 2023 Jan 4;6(1):e2249353. doi: 10.1001/jamanetworkopen.2022.49353 (PMC9857618; doi:10.1001/jamanetworkopen.2022.49353)
Supplement: Supplement 2. — Data Sharing Statement [file jamanetwopen-e2249353-s002.pdf]

Data Sharing Statement

Ohnuma. Association of Appropriate Empirical Antimicrobial Therapy With In-Hospital Mortality in Patients With Bloodstream Infections in the US. *JAMA Netw Open*. Published January 04, 2023. doi:10.1001/jamanetworkopen.2022.49353

Data
Data available: No
